# Occult extraperitoneal rectal injury and necrotizing fasciitis of the thigh following traumatic pelvic fracture: case report

**DOI:** 10.1093/jscr/rjag1015

**Published:** 2026-11-02

**Authors:** Murad Alahmad, Fernando Spencer Netto, Ibrahim Afifi, Ahmed F Ramzee, Ruben Peralta, Mohamed H Ahmed, Ayman El-Menyar, Hassan Al-Thani, Sandro Rizoli

**Affiliations:** Department of Trauma Surgery, Hamad Medical Corporation, Al Rayyan Street, PO Box 3050, Doha, Qatar; Department of Trauma Surgery, Hamad Medical Corporation, Al Rayyan Street, PO Box 3050, Doha, Qatar; Department of Trauma Surgery, Hamad Medical Corporation, Al Rayyan Street, PO Box 3050, Doha, Qatar; Department of Trauma Surgery, Hamad Medical Corporation, Al Rayyan Street, PO Box 3050, Doha, Qatar; Department of Trauma Surgery, Hamad Medical Corporation, Al Rayyan Street, PO Box 3050, Doha, Qatar; Department of Surgery, Universidad Nacional Pedro Henriquez Urena, Santo Domingo 10100, Dominican Republic; Department of Medical Education, Hamad Medical Corporation, Doha, Qatar; Department of Trauma Surgery, Clinical Research, Hamad Medical Corporation, Doha, Qatar; Department of Clinical Medicine, Weill Cornell Medicine, Education City, PO Box 24144, Doha, Qatar; Department of Trauma Surgery, Hamad Medical Corporation, Al Rayyan Street, PO Box 3050, Doha, Qatar; Department of Trauma Surgery, Hamad Medical Corporation, Al Rayyan Street, PO Box 3050, Doha, Qatar

**Keywords:** extraperitoneal, rectal injury, pelvic fracture, necrotizing fasciitis

## Abstract

Extraperitoneal rectal injuries following blunt trauma are rare and often difficult to diagnose. In the setting of pelvic fractures, these injuries may be missed on initial imaging, leading to severe complications. We report a 40-year-old male who sustained a pelvic crush injury. Computed tomography (CT) demonstrated complex pelvic fractures with no evidence of rectal injury. Subtle extraperitoneal perirectal air was identified on further imaging. On day 13, he developed sepsis without an obvious source. Repeat CT revealed an extraperitoneal rectal perforation with extensive air tracking into the pelvic soft tissues and thigh. The patient underwent emergent surgical intervention including loop colostomy, proctoscopy, and extensive debridement for necrotizing soft tissue infection (NF). He subsequently improved with staged debridement and negative pressure wound therapy. This case highlights the diagnostic challenges of occult extraperitoneal rectal injuries. Unexplained extraperitoneal air should prompt further evaluation with endoscopy. Delayed diagnosis resulted in life-threatening complications e.g. NF.

## Introduction

Pelvic fractures are associated with significant morbidity and mortality, largely due to the high incidence of concomitant injuries and overall injury severity [1]. Rectal injuries are uncommon following blunt trauma but occur more frequently in the setting of high-energy pelvic fractures, particularly those involving disruption of the posterior pelvic ring or symphysis pubis [2, 3].

Extraperitoneal rectal injuries pose a significant diagnostic challenge. Clinical findings are often subtle or absent, and although computed tomography (CT) is a cornerstone of trauma evaluation, its sensitivity for detecting early or subtle gastrointestinal injuries remains limited [4]. Delayed or missed diagnosis may result in severe complications, including pelvic sepsis and, in rare cases, necrotizing soft tissue infection (NF) [5].

Direct endoscopic evaluation, including proctoscopy and rigid sigmoidoscopy, remains an essential adjunct in the assessment of suspected rectal injury, particularly when CT findings are inconclusive [6]. We report a rare case of occult extraperitoneal rectal injury following pelvic fracture presenting as NF of the thigh, highlighting diagnostic pitfalls and management considerations.

## Case presentation

A 40-year-old male with a history of type II diabetes mellitus, managed with oral hypoglycemic agents, presented following a pelvic crush injury sustained when an electric gate closed on him, resulting in prolonged compression. On arrival, he was alert, hemodynamically stable, and complained of left-sided pelvic pain. A pelvic binder was in place. Primary survey was unremarkable. Secondary survey revealed superficial abdominal abrasions, left-sided tenderness, and blood at the urinary meatus. Neurological examination demonstrated decreased sensation in the L4–L5 dermatomes with right-sided foot drop. Distal pulses were intact, and an ankle–foot orthosis was applied. Initial radiographs demonstrated a displaced left iliac fracture, a comminuted right sacral fracture, and diastasis of the symphysis pubis. Plain X-ray and CT of the pelvis confirmed these findings and demonstrated multiple extraperitoneal pelvic and abdominal hematomas without evidence of active contrast extravasation (Fig. 1). No rectal or bowel injury was identified on initial imaging. Due to urethral bleeding and failed urethral catheterization, a suprapubic catheter was placed under ultrasound guidance, draining clear urine. A micturating cystourethrogram confirmed a bulbar urethral injury near the bulbomembranous junction. The patient was admitted to the Trauma Intensive Care Unit for close monitoring. On hospital day 2, the hemoglobin level dropped and repeated CT scan demonstrated interval enlargement of pelvic hematomas without evidence of active bleeding. The CT also revealed subtle extraperitoneal air in the perirectal region, which was attributed to recent suprapubic catheter insertion given the absence of symptoms and preserved bowel function (Fig. 2).

**Figure 1:**
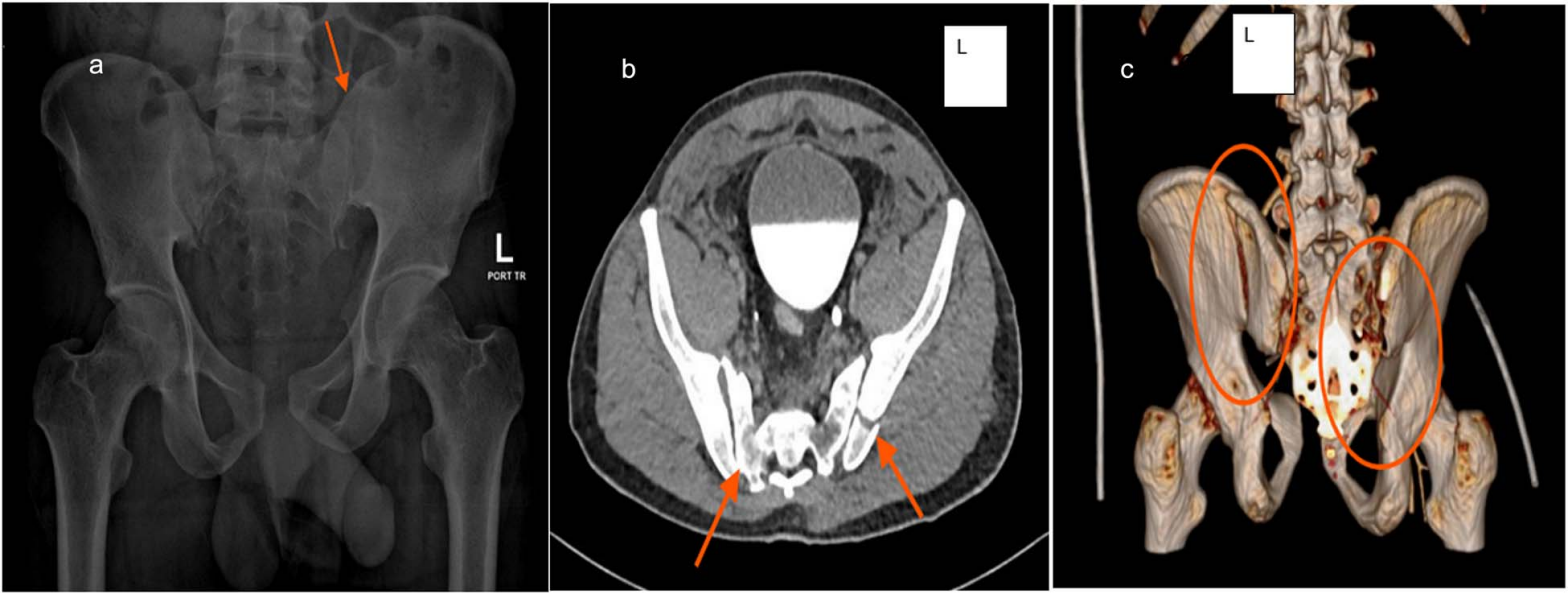
Plain X-ray (a) and CT scan of the pelvis (b, c) showing a displaced left iliac fracture, a comminuted right sacral fracture, and diastasis of the symphysis pubis.

**Figure 2:**
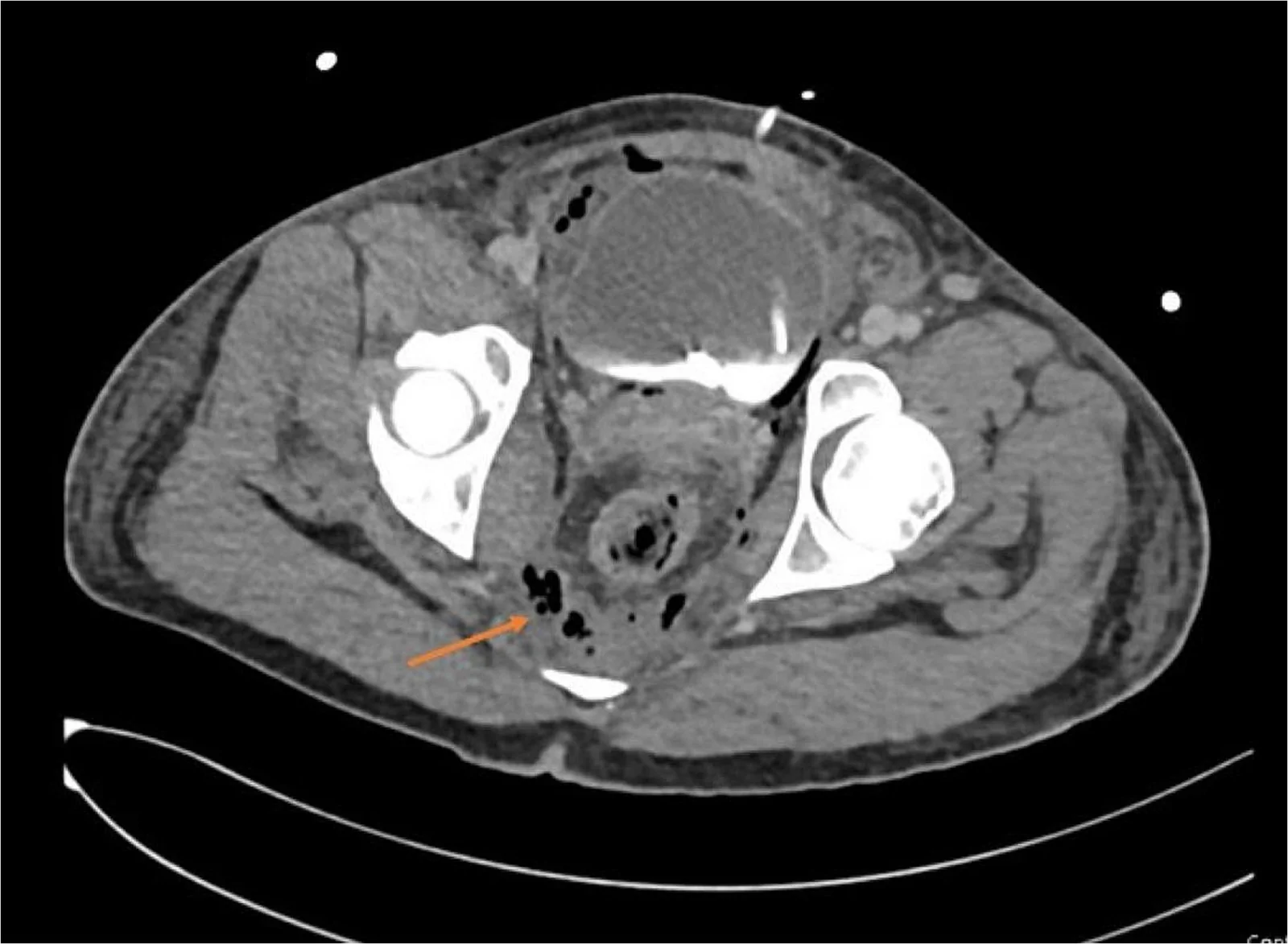
CT scan extraperitoneal air in the perirectal region, which was attributed to recent suprapubic catheter insertion.

On day 3, the patient underwent posterior sacroiliac screw fixation with anterior external pelvic fixation (Fig. 3). Postoperatively, he tolerated a full diet, maintained normal bowel function, and participated in physiotherapy for lower limb weakness.

**Figure 3:**
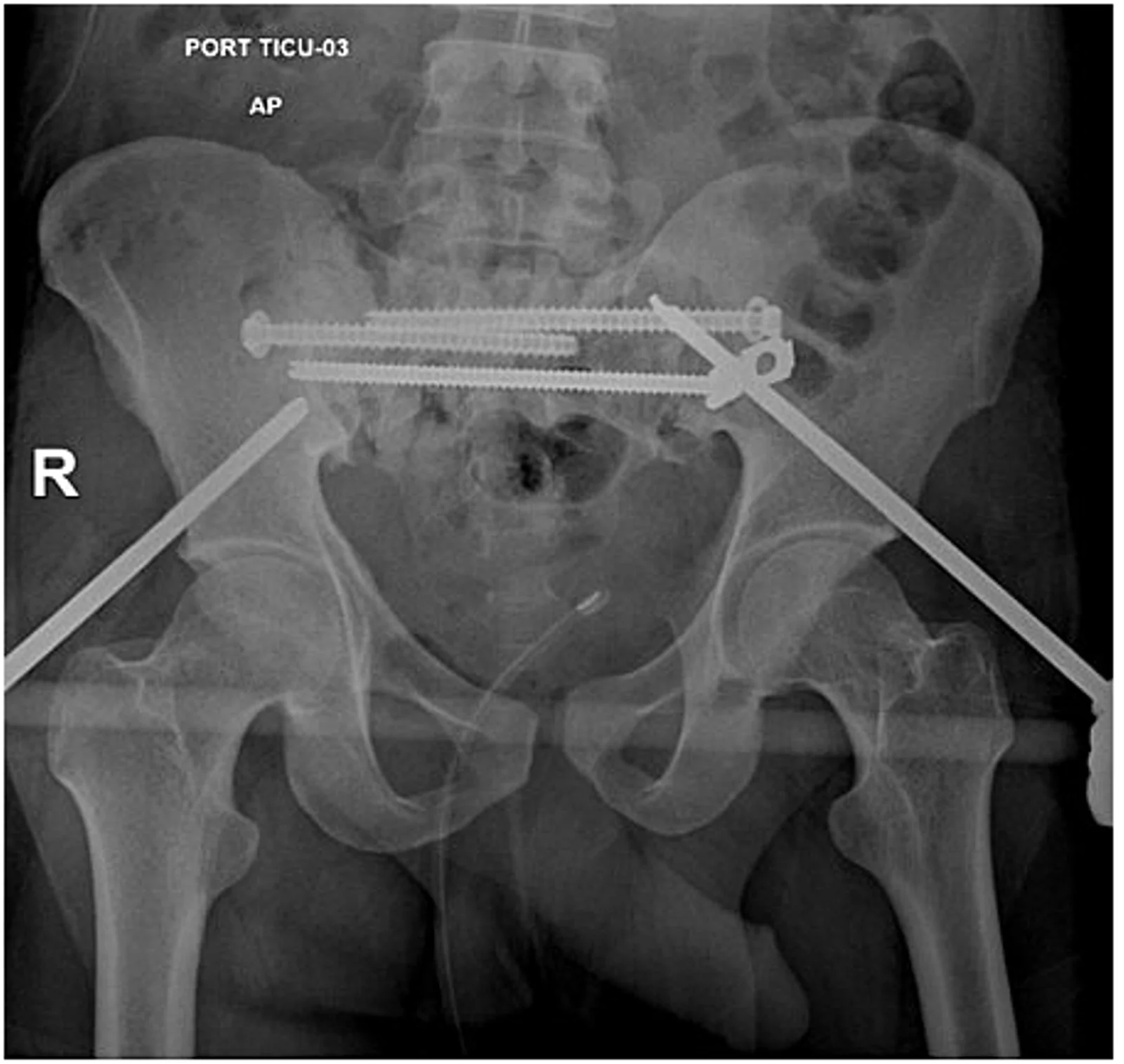
Pelvis X-ray showing posterior sacroiliac screw fixation with anterior external pelvic fixation.

On day 13, the patient developed sepsis without an identifiable source. Blood cultures revealed *Escherichia coli*, and broad-spectrum intravenous antibiotics were initiated. Despite antimicrobial therapy, he remained in a septic condition. Clinical examination revealed crepitus over the right thigh. Urgent CT scan revealed an extraperitoneal rectal perforation with extensive air tracking along the pelvic floor into the anterior abdominal wall, right inguinofemoral region, and right thigh, associated with multiple fluid collections (Fig. 4).

**Figure 4:**
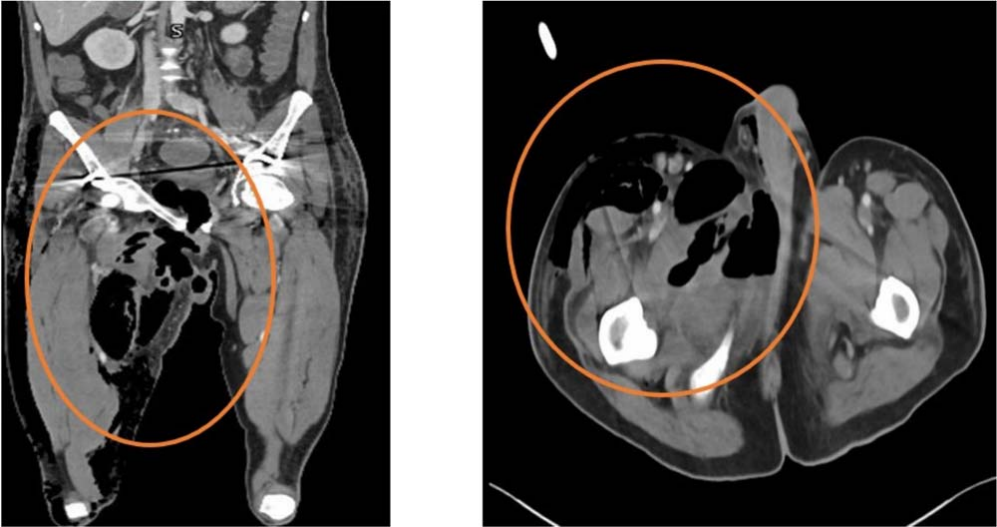
Coronal and transverse CT scan showing extraperitoneal rectal perforation with extensive air tracking along the pelvic floor into the anterior abdominal wall, right inguinofemoral region, and right thigh.

The patient emergently underwent laparoscopic exploration, loop colostomy formation, proctoscopy, and right thigh exploration. Proctoscopy identified an approximately 3 cm anterior rectal mucosal defect located 4 cm from the anal verge, communicating with a cavity extending toward the right thigh (Fig. 5). Exploration of the right thigh revealed extensive soft tissue NF with purulence, gas, and necrotic muscle, requiring aggressive debridement (Fig. 6).

**Figure 5:**
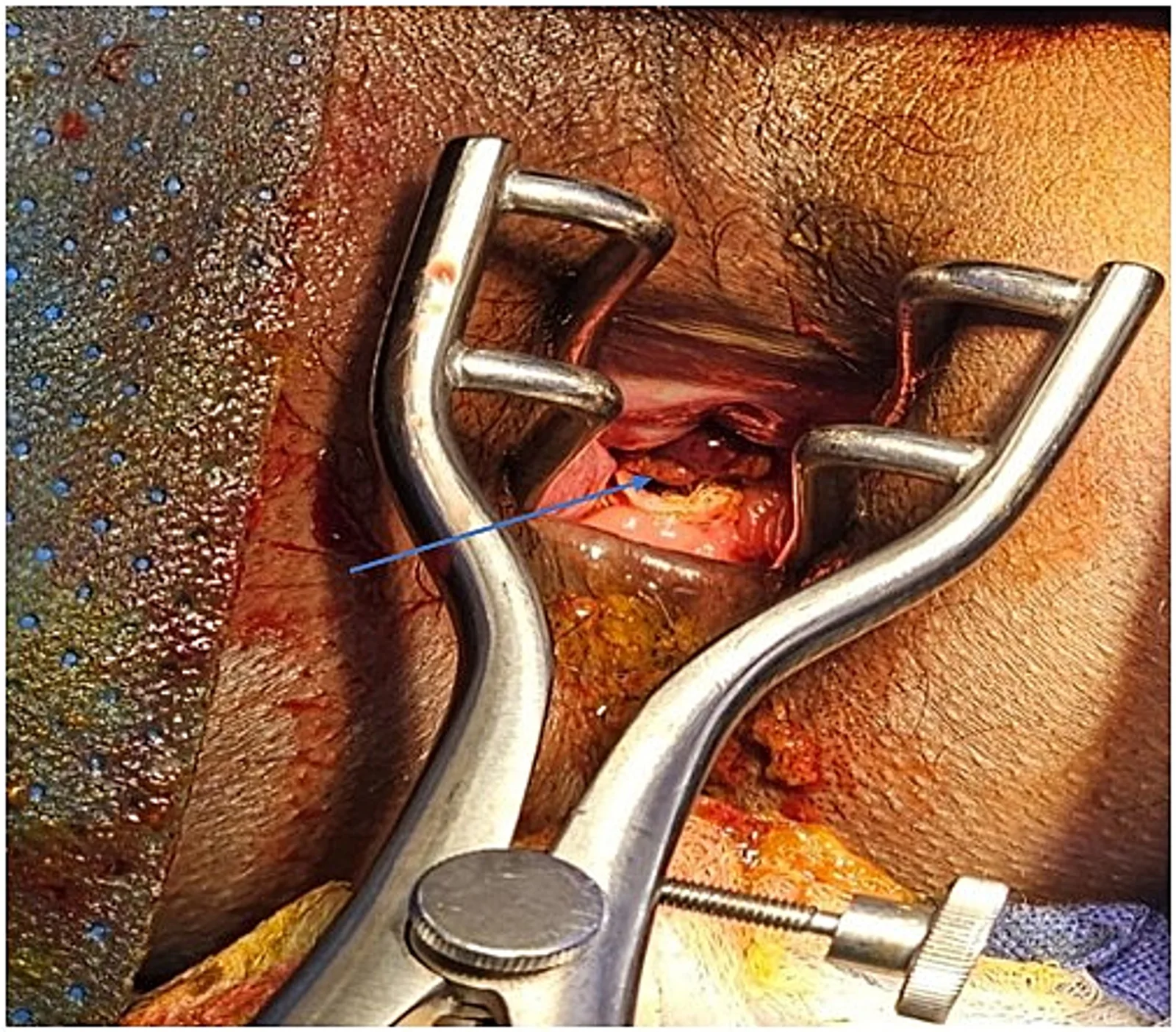
Rectal perforation: 3 cm anterior rectal mucosal defect located 4 cm from the anal verge.

**Figure 6:**
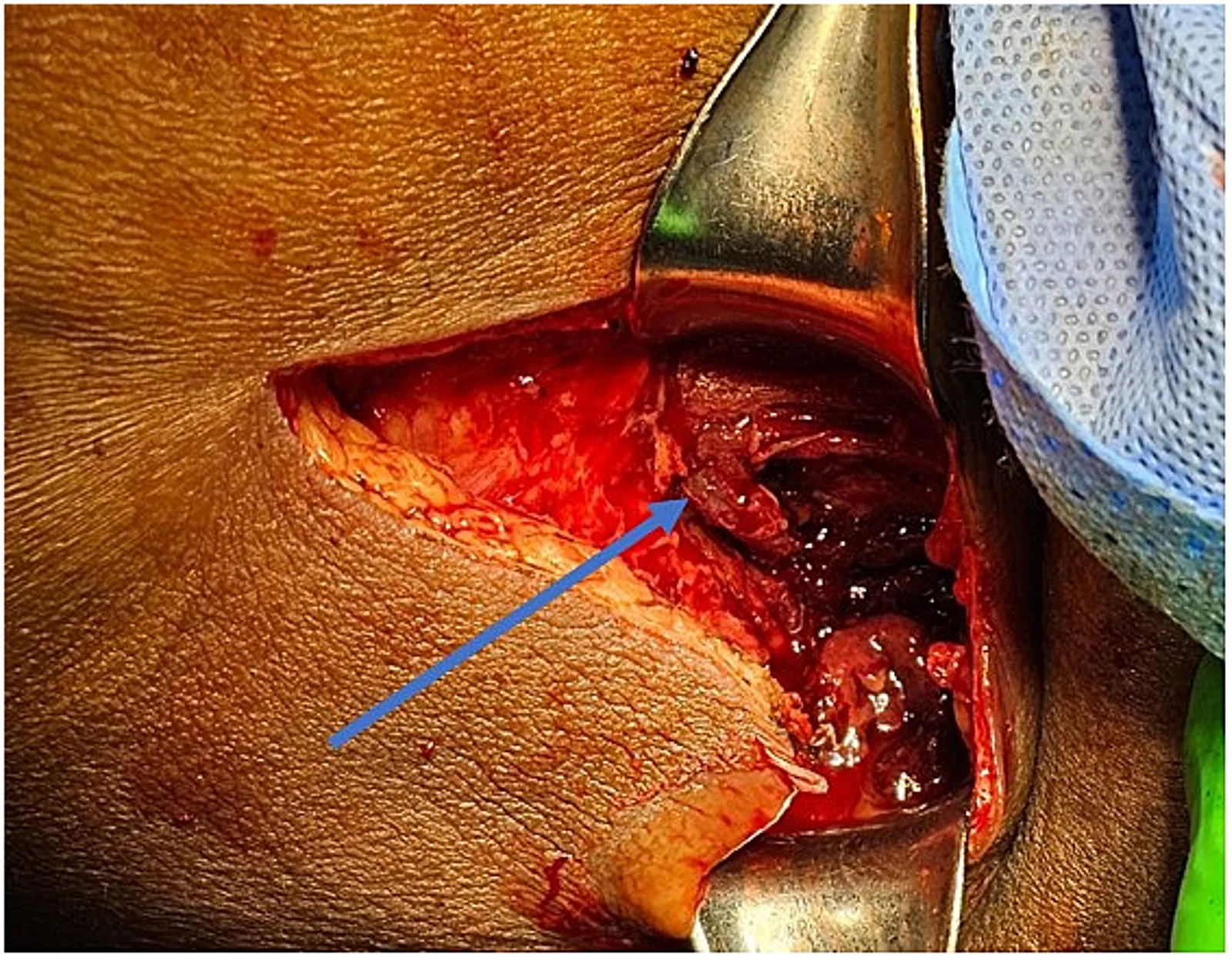
Right thigh exploration: Right extensive necrotizing soft tissue infection with purulence, gas, and necrotic muscle, communicating with the rectal perforation.

Postoperatively, he required a second debridement. The thigh wound was managed with negative pressure wound therapy and allowed to heal by secondary intention. The colostomy functioned appropriately, and the patient demonstrated gradual improvement. The hospital course was uneventful, and he was discharged in stable condition with a well-functioning stoma, with plans for future colostomy reversal and staged urethroplasty.

## Discussion

Rectal injuries are uncommon but carry significant morbidity, particularly when diagnosis is delayed [2]. In the setting of pelvic fractures, patterns involving the posterior pelvic ring, sacrum, and symphysis pubis have been associated with an increased risk of colorectal injury [3]. Although CT remains the cornerstone of trauma imaging, its sensitivity for detecting gastrointestinal injuries is limited, particularly in small or early perforations that may be obscured by surrounding hematoma or soft tissue injury [4]. In rectal trauma, CT alone may fail to identify a substantial proportion of injuries, and indirect findings such as extraperitoneal air can be difficult to interpret in complex pelvic fractures [7]. These limitations support the use of adjunctive diagnostic modalities. Endoscopic evaluation plays a crucial role in diagnosis. Rigid sigmoidoscopy allows full rectal visualization and is recommended when suspicion persists, while proctoscopy remains a valuable and readily available tool for distal extraperitoneal injuries [6, 8]. In this case, the lesion’s distal location allowed direct visualization with proctoscopy.

Early fecal diversion remains a cornerstone of management and is recommended to reduce pelvic sepsis and improve outcomes in extraperitoneal rectal injuries [9]. The delayed presentation as necrotizing fasciitis of the thigh is rare but anatomically plausible, as infection may track along pelvic fascial planes into the inguinofemoral region and thigh compartments [8]. Timely recognition and intervention are critical in soft tissue NF [10]. Delays in diagnosis have been associated with increased morbidity and adverse outcomes. This case underscores the importance of repeated clinical assessment, maintaining a high index of suspicion, and early endoscopic evaluation in patients with unexplained sepsis following pelvic trauma.

## Conclusion

Occult extraperitoneal rectal injury is a rare but potentially devastating complication of pelvic fractures. A negative CT scan does not reliably exclude rectal injury. Inconclusive imaging findings, including unexplained extraperitoneal or perirectal air in the absence of external wounds, should prompt further evaluation with proctoscopy or endoscopic assessment. Delayed diagnosis may result in atypical and life-threatening complications, including necrotizing fasciitis. Early recognition, timely fecal diversion, and aggressive surgical management are essential to improve patient outcomes.

## Data Availability

No new data were created or analyzed in this study.
